# Ileal and cecal microbiota response to *Salmonella* Typhimurium challenge in conventional and slow-growing broilers

**DOI:** 10.3389/fphys.2022.971255

**Published:** 2022-10-04

**Authors:** Tessa R. Sheets, Carmen L. Wickware, Ashlyn M. Snyder, Shawna L. Weimer, Timothy A. Johnson

**Affiliations:** ^1^ Department of Animal Sciences, Purdue University, West Lafayette, IN, United States; ^2^ Department of Animal and Avian Sciences, University of Maryland, College Park, MD, United States

**Keywords:** breed, poultry, 16S rRNA, slow-growing, colonization

## Abstract

Despite the negative impacts of *Salmonella* intestinal colonization on human health, *Salmonella* is a natural colonizer of the gastrointestinal tract and is not overtly pathogenic to the avian host. It is of interest to understand the impacts and colonization rates of *Salmonella* across selected genetic lines such as slow-growing (SG) and conventional (CONV) broilers. The objective of this study was to characterize the relationship between *Salmonella enterica* serovar Typhimurium challenge and selected broiler genetic lines on the ileal and cecal microbiome. Male chicks of two broiler breeds (*n* = 156/breed) were cohoused in an open floor pen until day 7. On day 13, the chicks were then separated into 12 isolators per breed (4 rooms, 6 isolators/room, 11 chicks/isolator). On day 14, chicks in the 12 treatment isolators (6 isolators/breed, 108 total) were challenged with *Salmonella* Typhimurium (ST) (1 × $10^{8}$ CFU/ml) via oral gavage while the remaining chicks (*n* = 108) were given an oral gavage of sterile tryptic soy broth control (C). Ileal and cecal contents were collected on day 7 from 24 chicks of each breed, and on days 13, 17, 21, and 24 from two chicks per isolator. Samples underwent DNA extraction and PCR amplification to obtain 16S rRNA amplicons that were sequenced with Illumina MiSeq. *Salmonella* Typhimurium colonization in the cecum was not different in the two broiler breeds. The main effect of breed had the greatest impact on the ileum microbiota of broilers 7 days of age where SG broilers had significantly lower diversity and richness compared to CONV broilers (*p* < 0.05). *Salmonella* Typhimurium challenge consistently caused a change in beta diversity. Regardless of day or intestinal location, challenged broilers had many amplicon sequence variants (ASVs) with decreased abundance of likely beneficial bacteria such as *Mollicutes* RF39, *Shuttleworthia*, *Flavonifractor*, and *Oscillibacter* compared to broilers that were unchallenged with *Salmonella* Typhimurium (*p* < 0.05). Additionally, there was a difference in the timing of when the microbiota alpha and beta diversity of each breed responded to *Salmonella* Typhimurium challenge. Thus, both broiler breed and *Salmonella* Typhimurium can impact the intestinal microbiota.

## Introduction

*Salmonella* is a prominent foodborne pathogen that is commonly present on broiler farms. Despite its negative public perception, *Salmonella* is a common gut colonizer in poultry, which may be why it is so ubiquitous in tainted meat products (Antunes et al., 2003). The strains that cause Salmonellosis in live broilers are different than the strains that are known to cause Salmonellosis in humans (Pieskus et al., 2006). However, since Salmonellosis is an important health concern, its effects are under constant surveillance and study to determine how *Salmonella* colonization can be limited in large broiler production systems (Finstad et al., 2012). The use of antimicrobials, the lack of genetic variability, and limited living space may all contribute to the colonization of poultry by *Salmonella* in commercial farms (Foley et al., 2011).

Concurrently, due to increased public concern for animal welfare, there is increased public interest in poultry products from slow-growing chickens. These broilers have been bred through less intensive genetic selection leading to slower growth and longer periods to reach market weight. Studies have shown that such breeds allow for better welfare outcomes, but as expected, there are differences in growth and efficiency when compared to conventional fast-growing broilers (Torrey et al., 2021). Further investigation is necessary to determine the response of a slow-growing line to pathogen exposure and colonization resistance. The role of broiler host genetic factors in resistance and immune response when exposed to *Salmonella* has been studied previously. For example, some breeds that were resistant to *S. enterica* serovar Typhimurium were also resistant to other serovars, including Gallinarum, Pullorum and Enteritidis (van Hemert et al., 2006). Additionally, different breeds allow varying levels of *Salmonella* colonization in the intestinal tract along with differing responses to vaccination (van Hemert et al., 2006). The intestinal microbiome of broilers may provide insight into colonization rate of *Salmonella* as well as the impact of host genetics on the composition of the microbiome. Recent advancements in bacterial identification and microbiome analysis by next generation sequencing (NGS) have determined that the development and microbial composition of the intestines are influenced by genetics (Zhao et al., 2013; Mignon-Grasteau et al., 2015). Microbiota are essential for maintaining a healthy gut and preventing colonization of pathogenic bacteria, while *Salmonella* colonization may lead to dysbiosis and increased susceptibility to disease. The microbiota most commonly found within the intestinal tract of broilers belong to the phyla Firmicutes, Bacteroidetes, and Proteobacteria, and consist of hundreds of genera responsible for aiding in absorption and digestion in the ileum and cecum, respectively (Oakley et al., 2014; Sergeant et al., 2014; Clavijo and Flórez, 2018). However, the gut microbiome is complex and variable due to differences in age, intestinal region, diet, and genetics. More research is needed to clearly identify the relationship between pathogen exposure, broiler genetics, and the resulting microbial community.

The purpose of this study was to evaluate differences between ileal and cecal microbiomes of conventional and slow-growing broilers when challenged with *Salmonella* Typhimurium. It was hypothesized that the microbiome would differ between breeds and challenge status, as selection for growth has been associated with lower resistance to *Salmonella* (Guillot et al., 1995; Kramer et al., 2003). Similar to other studies, it was also expected that broiler age would have a prominent effect on microbial community characteristics (Schokker et al., 2021). This work could aid in understanding how intensive selection has played a role in the development of the intestinal microbiome as well as identifying and managing the prevalence of *Salmonella* in broiler flocks.

## Materials and methods

### Animals and experimental design

All procedures were approved by the University of Maryland Animal Care and Use Committee (IACUC#: R-NOV-19-55). A 2 × 2 split plot design was utilized with 156 male slow-growing broilers (SG) and 156 male conventional broilers (CONV). On day 0, all 312 chicks were placed in an open floor pen for co-mingling to establish a baseline microbial community. On day 7, twenty-four chicks from each breed were euthanized to obtain ileal and cecal contents (*N* = 48 total birds). After sampling, the remaining chicks were transferred to an animal biosafety level (ABSL) 2 research facility and separated into six isolators in four rooms with 11 chicks in each isolator (*N* = 24, total isolators). *Salmonella* Typhimurium strain #289-1 (Cox and Blankenship, 1975) was utilized to challenge the selected broilers because it was nalidixic acid (NAL)-resistant to allow for its isolation from any naturally colonizing *Salmonella* spp. On day 14, two rooms were randomly selected, and all birds in those rooms were orally gavaged with 1 ml of a tryptic soy broth (TSB) culture containing 1.3 × 10^8^ colony forming units (CFUs)/ml *Salmonella* Typhimurium (ST) while birds in the other two rooms were orally gavaged with a saline control (C) of tryptic soy broth. Two chicks from each isolator were euthanized and their ileal and cecal contents were collected on days 13, 17, 21 and 24 (*N* = 48 samples per day). The contents for day 7 remained in one tube with no media to be used for DNA extraction. The contents for days 13–24 were divided between two tubes, one tube with glycerol to be used for bacterial culturing and one tube with no media to be used for DNA extraction. The tubes were shipped on dry ice to Purdue University where they remained at −20  ° C until further processing.

### *Salmonella* enumeration

All samples for enumeration of *Salmonella* Typhimurium were kept at −20 ° C in glycerol until used. Samples were plated in triplicate on Bismuth Sulfite agar, supplemented with 200 μg/ml nalidixic acid and incubated for 18–20 h at 37 ° F. To determine the initial dilution in 20% glycerol at the time of sample collection, the samples were centrifuged 4,000 rpm for 15 min to pellet the cecal contents and the volume of the pellet and solution was determined in milliliters. Samples were resuspended before returning to the freezer.

### Microbiome library preparation and analysis

DNA was extracted from samples using the MagAttract PowerMicrobiome DNA/RNA Kit (Qiagen, Hilden, Germany) following the manufacturer’s protocol. The concentration of the extracted DNA was determined using the Quant-iT PicoGreen dsDNA Assay Qubit dsDNA Assay Kit (Thermofisher Scientific Waltham, MA, United States) and subsequently normalized to 10 ng/ul by dilution in DNA-free molecular grade water. Extracted DNA was used for the construction of a 16S rRNA gene library following a standardized protocol (Kozich et al., 2013). Briefly, Illumina indexed amplicons were created using PCR amplification of the V4 region of bacterial 16S rRNA gene using the 515R (GTGCCAGCMGCCGCGGTAA)/806R (GGACTACHVGGGTWTCTAAT) primers. PCR and sequencing quality were assessed by preparing 16S rRNA gene libraries for a known positive control mock community (20 Strain Even Mix Genomic Material; ATCC® MSA1002TM) and water as a negative control. Amplification products were visualized through gel electrophoresis. No bands were observed in the negative control samples. Amplified DNA was normalized using a SequalPrep Normalization Plate (Invitrogen) and pooled into libraries containing the amplification products from 94 samples, mock community, and water. These libraries were sequenced (Illumina, MiSeq v2 kit, 2 × 500 cycle) at the Purdue Genomics Core Facility.

### 16S rRNA amplicon sequence analysis

Raw reads (25,694,292 ileal read pairs and 24,069,503 cecal read pairs) were analyzed using Quantitative Insight into Microbial Ecology (QIIME2, v.2020.2) (Bolyen et al., 2019). The general pipeline for QIIME2 is as follows: demultiplex samples from raw reads, process sequences through quality control filtering (DADA2) to remove low quality reads (Callahan et al., 2016), construct a feature table from corrected sequence data, produce a phylogenetic tree (Price et al., 2010), subsample features (max depth of 15,000 sequences per sample), and calculate diversity metrics. For DADA2, the 5’ end of the ileal forward and reverse sequences were not trimmed (--p-trim-left-f 0 and --p-trim-left-r 0) while they were truncated at position 251 (--p-trunc-len-f 251 and --p-trunc-len-r 251). For the cecal sequences, both forward and reverse reads were trimmed at position 5 (--p-trim-left-f 5 and --p-trim-left-r 5) and truncated at position 251. After removal and processing, a total of 18,173,050 high quality sequences were obtained from the ileum and 16,915,543 were obtained from the cecum for downstream analysis. Ileal data was subsampled to 32,448 sequences per sample, resulting in removal of four samples; one from day 7 and three from day 13. All were CONV broiler samples. Cecal data was subsampled to 20,689 sequences per sample, only removing one sample from a challenged CONV broiler on day 21.

### Data organization

In order to account for the effect of environment and age, samples from each day were analyzed separately resulting in five data sets. 7 day old chicks were co-housed together to establish a community baseline within the gastrointestinal tract. On day 7, chick was the experimental unit. After sequence quality filtering, 48 cecal and 40 ileal samples remained in the dataset. After day 7, the isolator was used as the experimental unit at each time point, so the counts of identical sequence groups (amplicon sequence variants, ASVs) of the two birds from the same isolator were averaged so that each bird was equally represented in the isolator composite sample. If one of the two replicates was removed during sequence quality filtering and rarefaction, the sequences from the remaining animal represented the isolator. In this study, all isolators on all time points had at least one representative animal.

### Statistical analysis

Statistics regarding *Salmonella* enumeration were completed using R software (v1.1.423). For each sample, the average colony forming unit (CFU) per milliliter cecal contents was calculated from the triplicate counts. A general linear model using ANOVA was created with the fixed effects of age and breed, and the random effect of isolator nested within room. Data were transformed using log10 to normalize the counts.

Alpha diversity metrics are used to describe community characteristics such as richness (observed ASVs), evenness (Pielou), and phylogenetic diversity (Faith) and biodiversity (Shannon). These were analyzed using a general linear model in R and a Type III Sum of Squares was utilized to account for unevenness between groups when running ANOVA. Assumptions for the normality of the residuals and homogeneity of variance were checked using the ggplot2 package and dependent variables not meeting these assumptions were log or square root transformed. Tukey’s test of additivity was utilized to determine if the interaction between the effect of genetic line and *Salmonella* challenge was statistically significant. Statistical significance was defined as *p* ≤ 0.05. Beta diversity (measure of dissimilarity between communities) was estimated using Bray Curtis, weighted Unifrac and unweighted Unifrac dissimilarities. Differences in beta diversity was determined using pairwise PERMANOVA tests. Differential abundance of genera was calculated with DESeq2 (v1.32.0) (Love et al., 2014). For purposes of reproducibility; metadata, scripts, and commands used in QIIME2 and R are available at https://github.com/sheets27/16SrRNABroilerSalmonella.

## Results

### *Salmonella* enumeration

**FIGURE 1:**
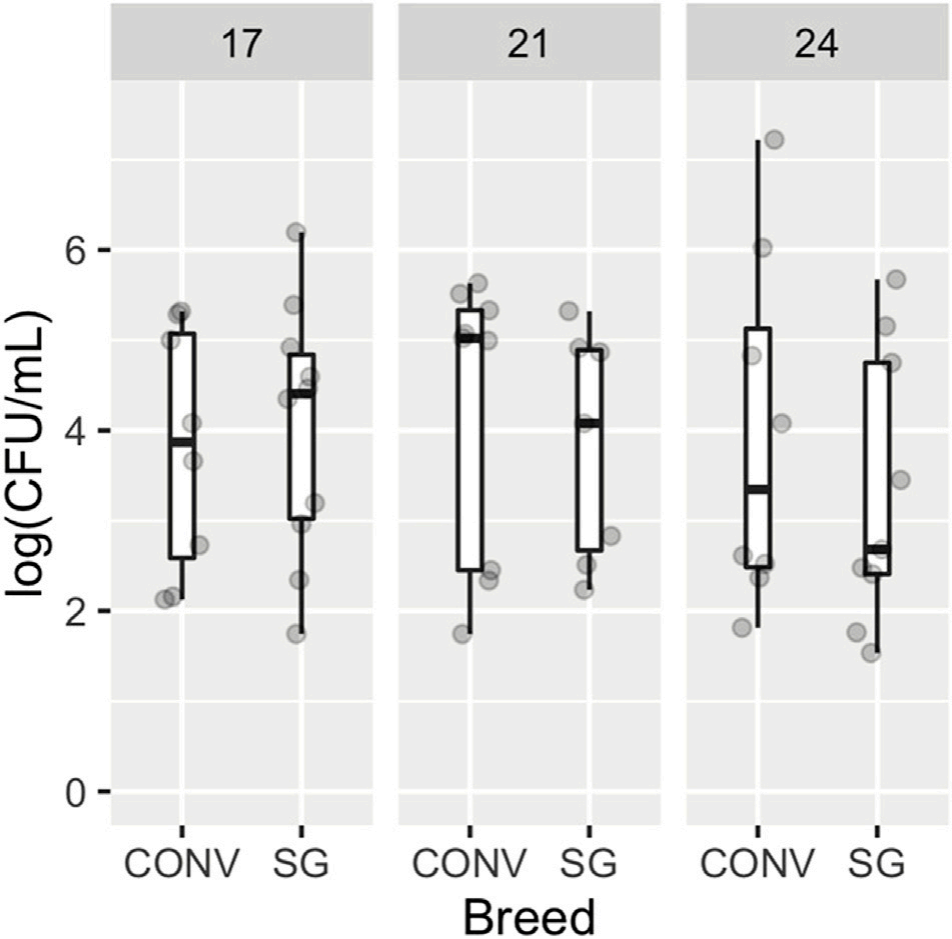
Enumeration of *Salmonella* Typhimurium from cecal contents of broilers. Box and whisker plots indicate the abundance of *Salmonella* Typhimurium in log colony forming units (CFU) per ml of cecal contents for conventional (CONV) and slow-growing (SG) broilers, separated by age. Points represent individual samples.

All samples from unchallenged birds were determined by plate count to not have nalidixic acid resistant *Salmonella* Typhimurium and thus are not part of the following analyses (data not shown). There were no significant differences in log (CFU/mL) of *Salmonella* Typhimurium due to age (*p* = 0.724, ω^2^ = −0.02) nor breed (*p* = 0.865, ω^2^ = −0.014) as shown in Figure 1. It should be noted that the omega squared value (ω^2^) indicates a small effect size. No interaction was found between age and breed.

### The impact of breed on the intestinal microbiome

**FIGURE 2:**
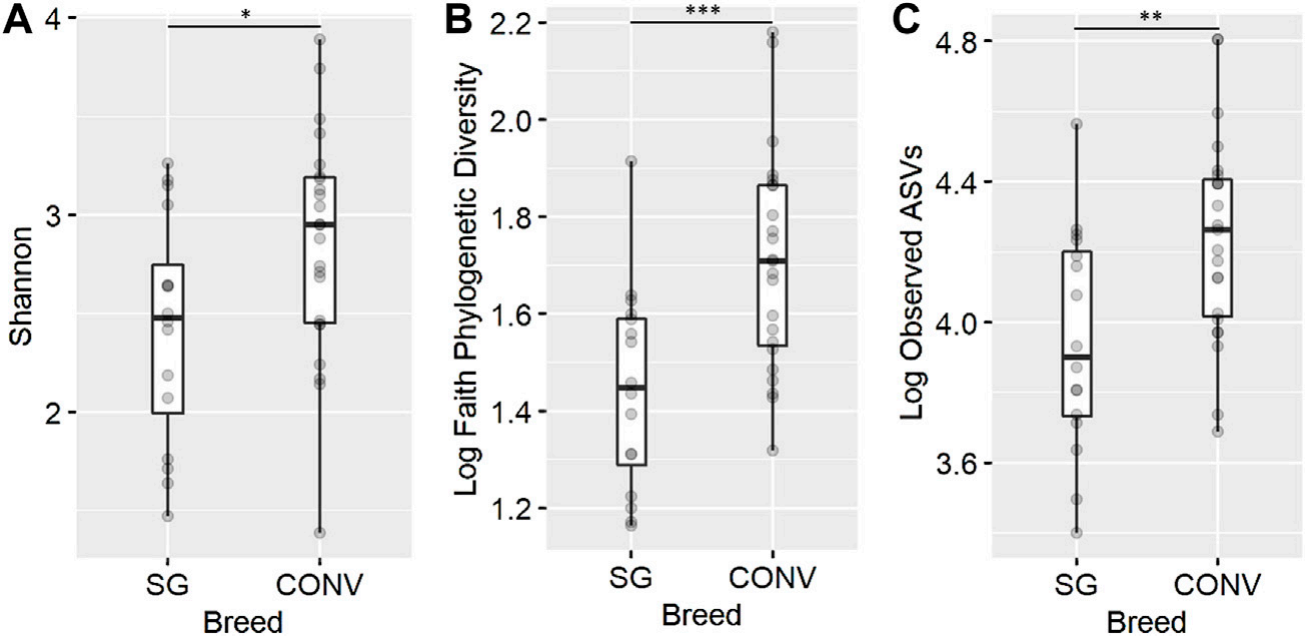
The effect of genetic line on the ileal Shannon diversity **(A)**, Faith phylogenetic diversity **(B)**, and observed ASV richness **(C)** for 7-day-old broilers. The presence of an asterisk indicates a significant difference between breeds in which * corresponds to *p* < 0.05, ** corresponds to *p* < 0.01, and *** corresponds to *p* < 0.001. Conventional (CONV) and slow-growing (SG) broilers.

In the ileum on day 7, conventional broilers (CONV) had significantly greater Shannon diversity, phylogenetic diversity (Faith), and richness (observed ASVs) compared to slow-growing broilers (SG) (Figure 2, ANOVA, *p* < 0.05). In the cecal contents on day 7, phylogenetic diversity, richness, and evenness (Pielou) remained the same between the two breeds. After separation into isolators and sampling on day 13, there was no significant difference in community alpha diversity between CONV and SG broilers when evaluating both intestinal region microbiomes. On days 17 and 21, Shannon diversity in the cecum was found to be significantly higher in SG compared to CONV (Supplementary Figure S1, ANOVA, *p* < 0.05). On day 24, the main effect of breed did not significantly impact alpha diversity in the ileum or cecum of broilers.

**FIGURE 3:**
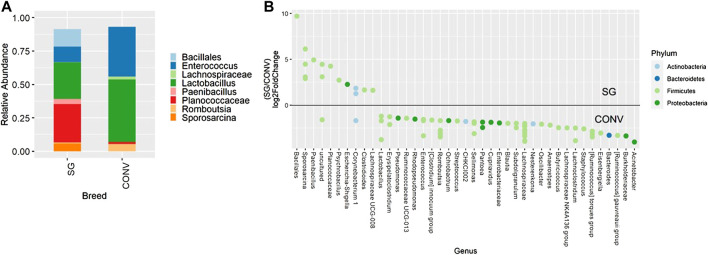
Relative abundance of genera in the ileum on day 7 that are present in greater than 2% of total taxa found in conventional (CONV) and slow-growing (SG) broilers **(A)**. Differentially abundant amplicon sequence variants (ASVs) between broiler genetic lines in the ileum on day 7 **(B)**. Significantly different (*p* < 0.05) ASVs are presented and organized by abundance within each breed. ASVs enriched in SG broilers are indicated with a log 2-fold change >0 while ASVs enriched in CONV broilers are indicated with a log 2-fold change of <0.

Beta diversity showed a similar pattern as alpha diversity for 7-day-old broilers. In the ileum samples on day 7, Bray-Curtis, unweighted Unifrac, and weighted Unifrac were all significantly affected by breed (Supplementary Figure S2, PERMANOVA, *p* < 0.05). Also in the ileum on day 7, ASVs assigned to *Enterococcus*, *Lactobacillus*, and *Romboutsia* were enriched in CONV broilers while ASVs assigned to *Bacillales*, *Sporosarcina*, *Paenibacillus*, and *Planococcaceae* were enriched in SG broilers (Figure 3, *p* < 0.05). In the cecum on day 7, Bray-Curtis distances were also slightly different between breeds (Supplementary Figure S3, PERMANOVA, *p* < 0.05) while Unifrac measures were not different. On day 13, the cecal communities in CONV and SG broilers were different when using Bray-Curtis and weighted Unifrac distances (Supplementary Figure S4, PERMANOVA, *p* < 0.05). An ASV assigned to *Lachnospiraceae* NK4A136 as well as ASVs assigned to *Oscillibacter*, *Ruminiclostridium* 9, and *Mollicutes* RF39, were enriched in the cecum of CONV broilers on day 13 (Supplementary Figure S5, *p* < 0.05). Similarly, Bray-Curtis and weighted Unifrac measurements were also significantly different because of breed when evaluating the cecum of 21-day-old broilers (Supplementary Figure S6, PERMANOVA, *p* < 0.05) with *Lachnospiraceae* NK4A136 being abundant in CONV broilers at this time point as well (data not shown).

### The impact of challenge on the intestinal microbiome

Several alpha diversity measures were affected by the main effect of *Salmonella* Typhimurium challenge. On day 17 of the experiment, 3 days after *Salmonella* gavage, challenged broilers (ST) had significantly lower Shannon diversity in the cecum compared to non-challenged, control broilers (C) (Supplementary Figure S7, ANOVA, *p* < 0.05). In regard to the ileum, ST broilers were found to have lower richness on day 21 but greater evenness on day 24 compared to controls (Supplementary Figure S8, ANOVA, *p* < 0.05).

**FIGURE 4:**
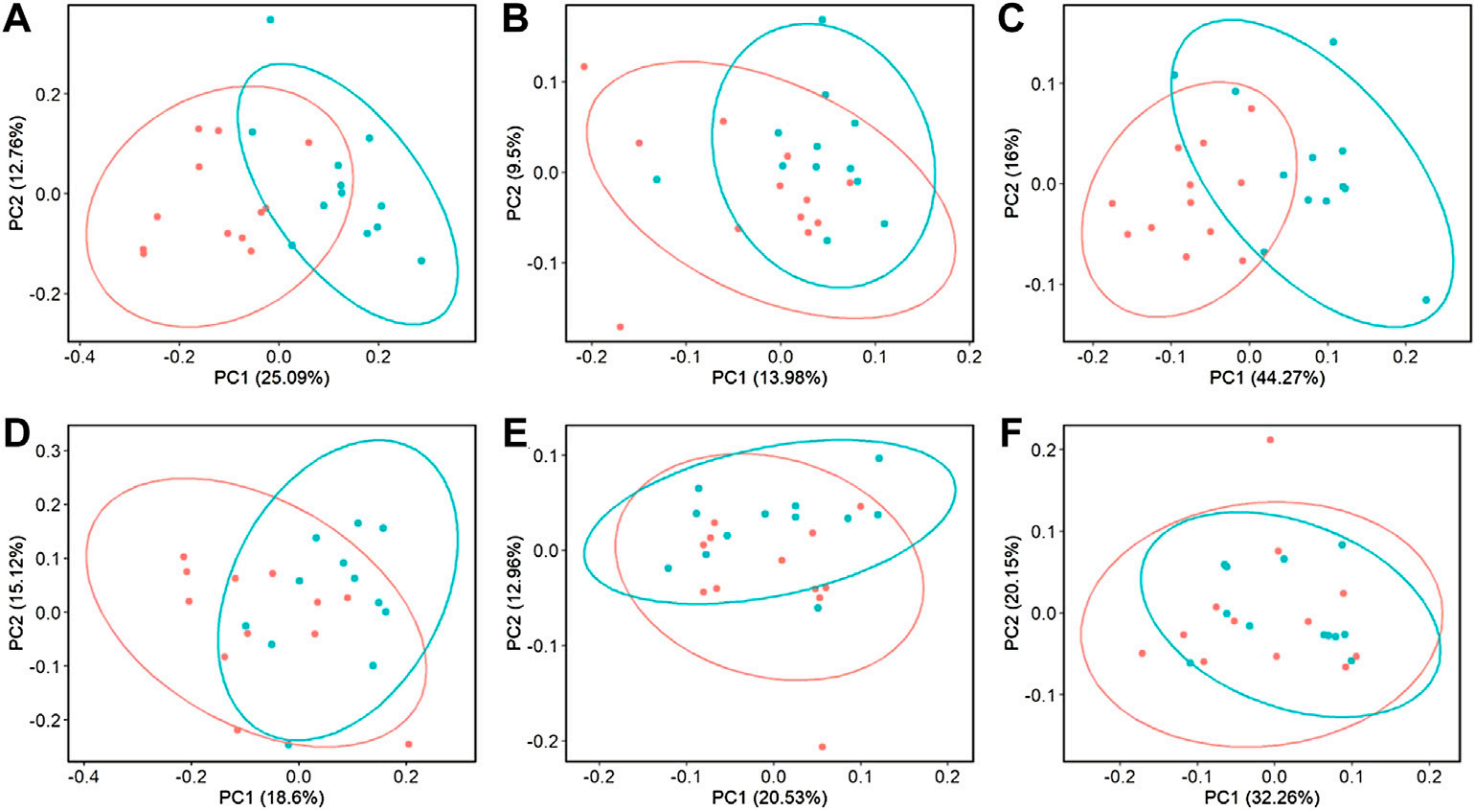
The effect of *Salmonella* challenge on beta diversity measures in the cecum. Significant dissimilarity was seen in Bray-Curtis **(A,D)**, unweighted Unifrac **(B,E)**, and weighted Unifrac **(C,F)** for 17 **(A–C)** and 24-day-old **(D–F)** broilers. Red represents control (C) broilers while blue represents *Salmonella* Typhimurium challenged (ST) broilers.

**FIGURE 5:**
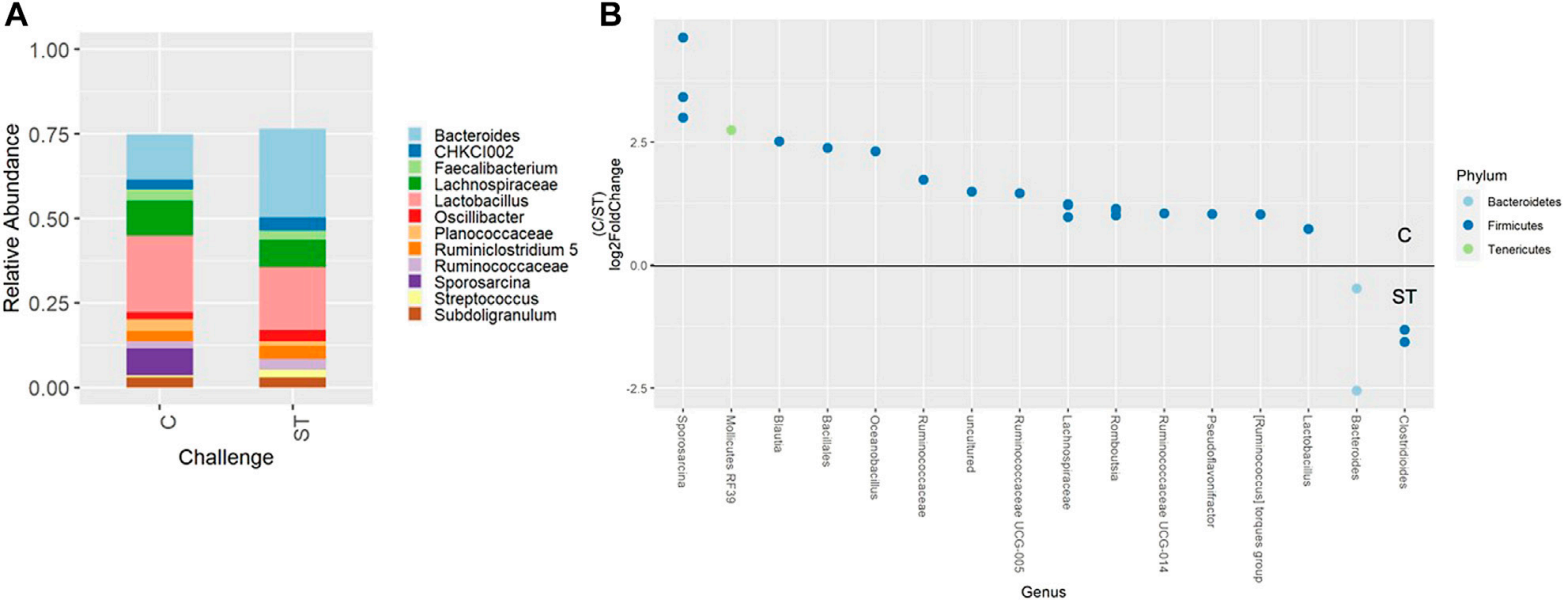
Relative abundance of genera in the cecum on day 17 that are present in greater than 2% of total taxa found within control (C) and *Salmonella* Typhimurium challenged (ST) broilers **(A)**. Differentially abundant amplicon sequence variants (ASVs) between broiler challenge groups in the cecum on day 17 **(B)**. Significantly different (*p* < 0.05) ASVs are presented and organized by abundance within each group. ASVs enriched in C broilers are indicated with a log 2-fold change >0 while ASVs enriched in ST broilers are indicated with a log 2-fold change of <0.

**FIGURE 6:**
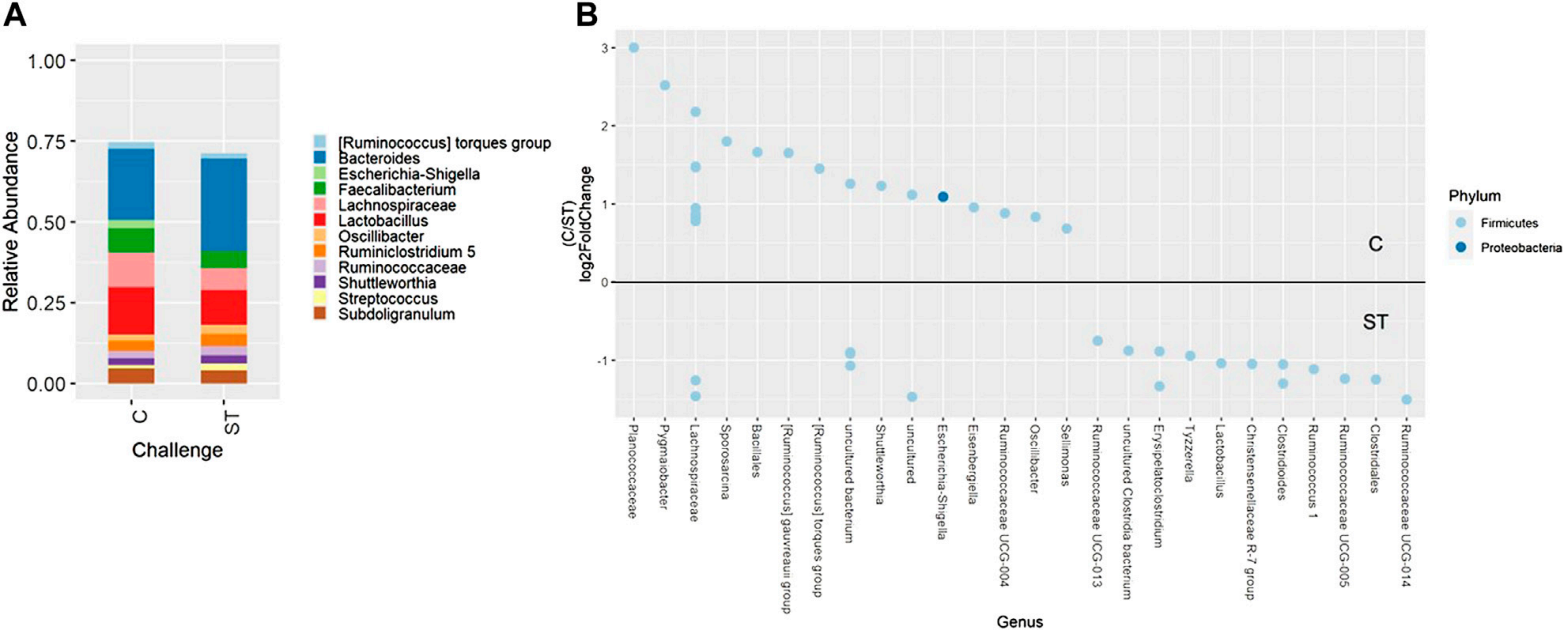
Relative abundance of genera in the cecum on day 24 that are present in greater than 2% of total taxa found within control (C) and *Salmonella* Typhimurium challenged (ST) broilers **(A)**. Differentially abundant amplicon sequence variants (ASVs) between broiler challenge groups in the cecum on day 24 **(B)**. Significantly different (*p* < 0.05) ASVs are presented and organized by abundance within each group. ASVs enriched in C broilers are indicated with a log 2-fold change >0 while ASVs enriched in ST broilers are indicated with a log 2-fold change of <0.

Beta-diversity analyses showed consistent cecal dissimilarity throughout later time points between ST and C broilers. There was substantial overlap in Bray-Curtis as well as unweighted and weighted Unifrac ellipses, yet these measures significantly differed due to challenge at 17 and 24 days of age (Figure 4, PERMANOVA, *p* < 0.05). Many differentially abundant genera were found when comparing the cecal communities of non-challenged to challenged broilers on both day 17 and 24. In challenged broilers on day 17, three *Sporosarcina* ASVs were reduced about 8-fold and two ASVs of *Bacteriodes* were increased (Figure 5, *p* < 0.05). On day 24, challenged broilers had decreased relative abundance of several ASVs including 5 *Lachnospiraceae* ASVs (decreased by 3-fold), and single ASVs of *Planococcaceae* (decreased by 8-fold), *Sporosarcina*, *Shuttleworthia*, *Ruminococcus gauvreauii* group, and *Oscillibacter* (Figure 6, *p* < 0.05).

**FIGURE 7:**
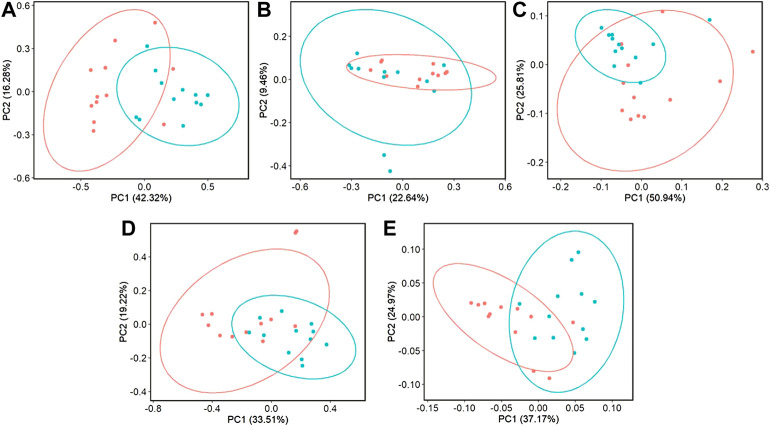
The effect of *Salmonella* challenge on beta diversity measures in the ileum. Significant dissimilarity was seen in Bray-Curtis **(A,D)**, unweighted Unifrac **(B)**, and weighted Unifrac **(C,E)** for 21 **(A–C)** and 24-day-old **(D,E)** broilers. Red represents control (C) broilers while blue represents *Salmonella* Typhimurium challenged (ST) broilers.

**FIGURE 8:**
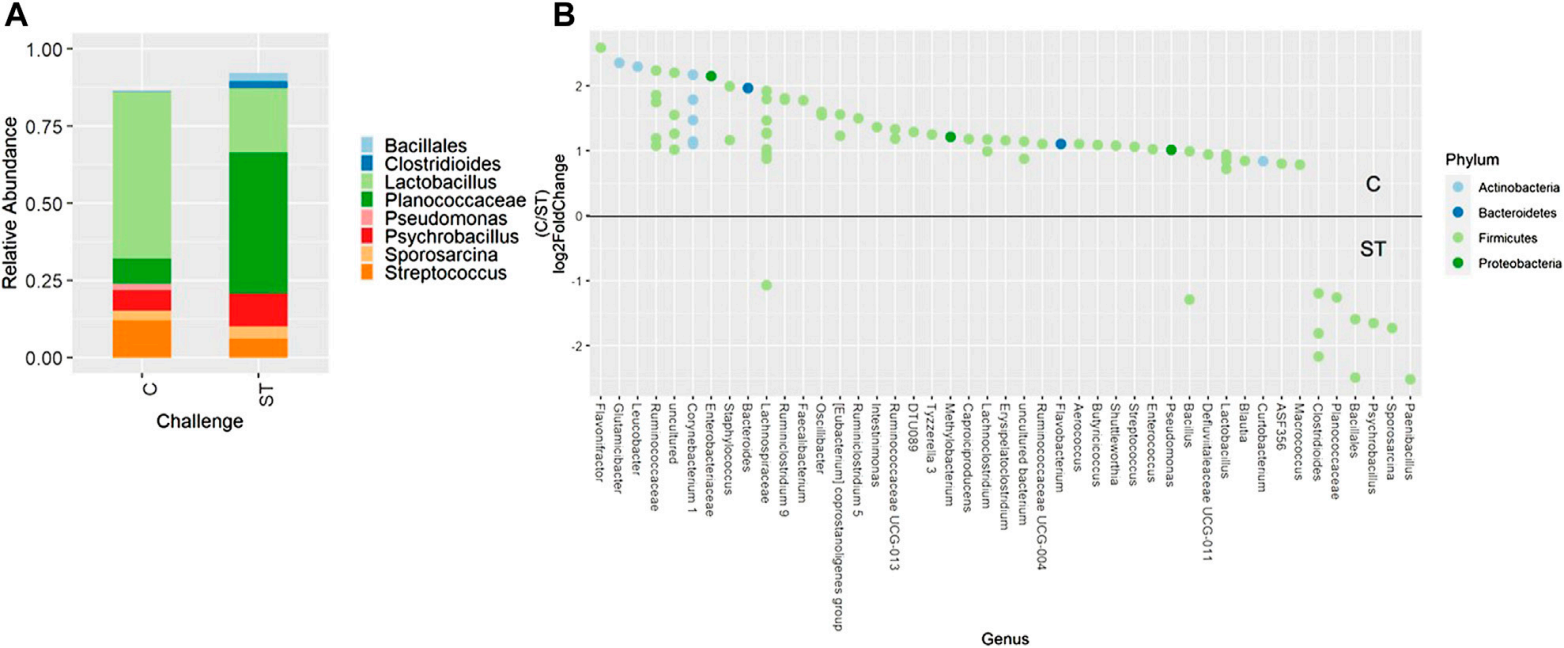
Relative abundance of genera in the ileum on day 21 that are present in greater than 2% of total taxa found within control (C) and *Salmonella* Typhimurium challenged (ST) broilers **(A)**. Differentially abundant amplicon sequence variants (ASVs) between broiler challenge groups in the ileum on day 21 **(B)**. Significantly different (*p* < 0.05) ASVs are presented and organized by abundance within each group. ASVs enriched in C broilers are indicated with a log 2-fold change >0 while ASVs enriched in ST broilers are indicated with a log 2-fold change of <0.

When evaluating ileal samples, all beta diversity measurements were found to be significantly different due to challenge on day 21 (Figures 7A–C, PERMANOVA, *p* < 0.05), and Bray-Curtis and weighted Unifrac distances were significantly different at day 24 (Figures 7D,E, PERMANOVA, *p* < 0.05). Along with community dissimilarity between broilers of different challenge states, many differentially abundant ASVs were identified. On day 21, ASVs from 37 genera were increased in non-challenged broilers, including *Flavonifractor*, *Ruminiclostridium* 9, *Oscillibacter*, and *Shuttleworthia* while challenged broilers were enriched in ASVs from only 6 genera including *Clostridioides*, *Bacillales*, and *Paenibacillus* (Figure 8, *p* < 0.05). Fewer differentially abundant genera were found in the ileum of 24 day old broilers, but *Clostridioides* continued to be differentially abundant in ST broilers compared to C broilers (Supplementary Figure S9, *p* < 0.05).

### The interaction effects between breed and *Salmonella* challenge on the intestinal microbiome

**FIGURE 9:**
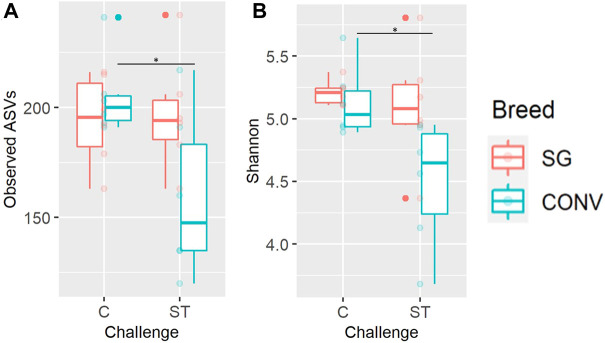
The interaction between of genetic line and *Salmonella* challenge on the cecal observed amplicon sequence variant (ASV) richness **(A)** and Shannon diversity **(B)** for 17-day-old broilers. The presence of an asterisk indicates a significant difference between pairwise groups in which * corresponds to *p* < 0.05, ** corresponds to *p* < 0.01, and *** corresponds to *p* < 0.001. Control (C) and *Salmonella* Typhimurium challenged (ST) broilers.

**FIGURE 10:**
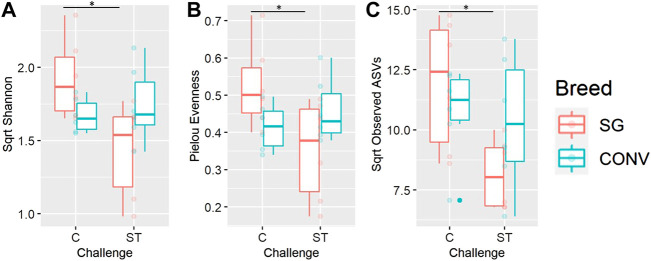
The interaction between of genetic line and *Salmonella* challenge on the ileal Shannon diversity **(A)**, Pielou evenness **(B)**, and observed amplicon sequence variant (ASV) richness **(C)** for 21-day-old broilers. The presence of an asterisk indicates a significant difference between pairwise groups in which * corresponds to *p* < 0.05, ** corresponds to *p* < 0.01, and *** corresponds to *p* < 0.001. Control (C) and *Salmonella* Typhimurium challenged (ST) broilers.

Alpha and beta diversity metrics of both the ileal and cecal microbiome were impacted by *Salmonella* challenge differently according to breed (interaction effect). In the ileum, there was a significant interaction between breed and challenge when measuring Shannon diversity, richness, and phylogenetic diversity of 7-day-old broilers, yet there were no significant pairwise comparisons when evaluating this interaction (Supplementary Figure S10, Tukey, *p* < 0.05). When evaluating the cecum on day 17, there was a significant breed and challenge interaction regarding richness (observed ASVs metric) with challenged CONV broilers having significantly lower richness compared to non-challenged CONV broilers (Figure 9A, Tukey, *p* < 0.05). No interaction was detected in Shannon diversity but challenged CONV broilers had significantly lower Shannon diversity compared to non-challenged CONV broilers in the cecum on day 17 (Figure 9B, Tukey, *p* < 0.05). On day 21 in the ileum, an interaction was detected where challenged SG broilers had significantly lower Shannon diversity, Pielou evenness, and observed ASV richness compared to non-challenged SG broilers (Figure 10, Tukey, *p* < 0.05). On day 21 and 24 in the cecum, only phylogenetic diversity exhibited a significant interaction between breed and challenge, with no significant pairwise comparisons for this alpha diversity measure (Supplementary Figure S11, Tukey, *p* < 0.05).

Regarding beta diversity, there was a significant interaction between breed and challenge on day 17 in the ileum when evaluating weighted Unifrac distances (Supplementary Figure S12, PERMANOVA, *p* < 0.05). Specifically, differences were found between CONV broilers of different challenge states and between challenged SG and challenged CONV broilers (Supplementary Figure S12, Pairwise PERMANOVA, *p* < 0.05). For the remaining days, no significant beta diversity interaction effects were found in ileal or cecal samples.

## Discussion

Ileal and cecal microbiomes have been characterized and well-studied in conventional broiler populations to help identify how modulations of the gut microbial community could influence a variety of performance and disease responses (Oakley et al., 2013; Stanley et al., 2013; Clavijo and Flórez, 2018). This is because the microbial community of the gastrointestinal tract plays a role in extracting energy from nutrients as well as harboring potential pathogenic organisms such as *Campylobacter*, *Salmonella enterica*, *Escherichia coli*, and *Clostridium perfringens* that can colonize and cause illness to the avian host and humans (Clavijo and Flórez, 2018). The current body of literature includes many microbiome analyses regarding the effect of probiotics (Gao et al., 2017; Rodrigues et al., 2020; Gyawali et al., 2022) or pathogen exposure (Park et al., 2017; Latorre et al., 2018; Joat et al., 2021) on the broiler microbiome. However, it should be noted that many of these single-facet studies utilize conventional, fast-growing broilers such as the Ross or Cobb breeds. Moreover, the concern for improved animal welfare has allowed slow-growing commercial broiler breeds to become recognized by well-informed consumers (Lusk, 2018). Previous research has compared characteristics between slow-growing and conventional broilers related to differences in behavior (Bokkers and Koene, 2003; Çavuşoğlu and Petek, 2019), gene profile (Cui et al., 2012; Hu et al., 2013), carcass traits (Mikulski et al., 2011; Singh et al., 2021), or immune response (Williams et al., 2013; Giles et al., 2019), while a limited number of studies have compared slow-growing and conventional breeds in their intestinal microbiota and the interaction of the microbiota with a separate factor such as the addition of a feed additive or pathogen challenge. Therefore, the purpose of this study was to investigate the ileal and cecal microbiota response to *Salmonella* challenge in both conventional and slow-growing broilers. The behavior and immune response results from the broilers in this study have been published previously (Snyder et al., 2022).

The impact of genetics on the development and composition of the intestinal microbiome is an active area of research in efforts to understand factors that control microbiome modulation. In the present study, there were significant differences in the alpha diversity of both the ileal and cecal microbiome of broilers, but these distinctions were variable across time and dependent on the intestinal location under study. The ileal microbial community alpha and beta diversities were affected by breed on day 7, while the effect of genetic line became prominent in the cecal microbial community beta diversity beginning on day 13. Differences in microbial composition appear to begin in the ileum and progress to the cecum due to differential development of the tissue between the two breeds during those times (Danzeisen et al., 2015). Strong selection for broilers with high digestive efficiency produced heritable microbial communities with specific ratios of bacteria such as *Lactobacillus crispatus*, *Clostridium leptum*, and *Clostridium coccoides*, and *Escherichia coli* (Mignon-Grasteau et al., 2015). The intimate relationship between bacteria and nutrient digestibility may allow for a clearer signal when evaluating the interaction between breed and microbial composition (Kim et al., 2015; Marmion et al., 2021). Additionally, one study used highly established genetic lines to determine that the abundance of 29 fecal microbiome species was different between two egg-laying chicken breeds after 54 generations of selection for high or low market body weight (Zhao et al., 2013). One review evaluating the ileal microbiota of Ross and Cobb broilers emphasized that these breeds have different microbiota compositions, yet taxonomical enrichment was not consistently present in either breed over time (Kers et al., 2018). The results presented in this study and others suggest that the gut microbiome can be distinct between two different broiler breeds and the level of this distinction may be influenced by the selected trait and the length of selection as well as age.

The clearest sign that breed affected broiler intestinal microbiome in the current study was at the youngest age. On day 7, slow-growing broilers had lower diversity and richness in the ileum compared to fast-growing, conventional broilers. Not only was a change in the ileal microbiome observed, but on the same day, conventional broilers were found to have greater villus height and crypt depth in the jejunum based on a concurrent study, suggesting better intestinal health (Snyder et al., 2022). Thus, the microbial communities between a slow-growing broiler and a conventional broiler may vary because of the difference in the morphology of the small intestines. The impact of breed on microbiota diversity measures became less clear in the ileum as the birds grew older and their intestinal morphology developed. Indeed, current literature shows age commonly has a greater impact on the alteration of the microbiome compared to breed (van der Wielen et al., 2002; Hume et al., 2003; Gong et al., 2008). This, together with our data, suggests that observing the direct impact of breed on the microbiome becomes more difficult as the host grows older and the microbiome becomes altered potentially due to intestinal tissue development and exposure to new environmental conditions (Díaz-Sánchez et al., 2019). This may be of importance in regard to identifying beneficial feed additives that have the purpose of affecting the microbiome. A more apparent response might be expected between different broiler breeds when the additive is administered at a younger age.

Differences seen in beta diversity between slow-growing and conventional broilers are further understood when examining differences in taxonomic profile. On day 7, several taxa from Firmicutes that have been shown to be advantageous were differentially abundant in slow-growing and conventional broilers. For example, only *Sporosarcina* was significantly enriched in slow-growing broilers and previous research has indicated that *Sporosarcina* may have potential probiotic characteristics (Priyodip and Balaji, 2019). On the other hand, conventional broilers in the current study were found to have a single amplicon sequence variant of *Oscillibacter*, *Lachnospiracea* NK4A136, and *Ruminococcus gauvreauii* that were differentially abundant in the ileum on day 7. Both *Oscillibacter* and *Lachnospiracea* NK4A136 are short chain fatty acid producers while *Ruminococcus gauvreauii* may be involved with feed efficiency (Madigan-Stretton et al., 2020).

Along with genetics and age, it was found that challenge with *Salmonella* Typhimurium caused community shifts in alpha and beta diversity in both the ileum and cecum. Beneficial microbes such as *Mollicutes* RF39, *Shuttleworthia*, *Ruminiclostridium* 9 and *Flavonifractor* were found to be decreased in the intestinal tract of challenged broilers in the cecum on day 17, the ileum on day 21, and in both intestinal locations on day 24. *Flavonifractor* is positively correlated with body weight and average daily gain in broilers given *Bacillus subtilis* as a probiotic (Zhang et al., 2021). *Shuttleworthia* may also contribute to nutrient absorption because it contributes to carbohydrate and lipid metabolic pathways (Chen et al., 2020). *Ruminiclostridium* 9 plays a role complex carbohydrate metabolism (Joat et al., 2021) and members from the Mollicutes class are involved with energy harvesting in the gastrointestinal tract (Turnbaugh et al., 2008). From our results, the introduction of *Salmonella* affected the relative abundance of certain commensals in the microbiome, resulting in a change in taxonomic profile and overall composition of the microbial community. This could be of significance because *Salmonella* challenge may be a contributing factor to dysbiosis or further bacterial invasion in the intestinal microbiota. These possible side-effects would need to be tested further to be confirmed.

The present study evaluated the interaction between genetic line and *Salmonella* challenge to find the intestinal microbiome was impacted differently by *Salmonella* between broilers of each breed at specific time points. These varying results have been found in several other studies including one that analyzed the outcome of *Campylobacter* infection between slow-growing and fast-growing broilers, finding fast-growing broilers infected with *Campylobacter jejuni* had greater incidences of pododermatitis compared to slow-growing breeds (Williams et al., 2013). Organic flocks composed of slow-growing broilers had a significantly higher prevalence of *Campylobacter* compared to broilers in conventional flocks (Heuer et al., 2001). Despite being colonized with *Salmonella* Typhimurium to the same extent, there were significant differences in how slow-growing and conventional broilers were impacted by *Salmonella* challenge. Alpha diversity measures decreased in the cecum of challenged conventional broilers on day 17 whereas alpha diversity measures deceased in the ileum of challenged slow-growing broilers on day 21. These results suggest that the microbiome of these two genetic lines are susceptible to *Salmonella*-induced dysbiosis at different times and intestinal locations. Further research is warranted to understand the specific immune response to *Salmonella* challenge in these broiler lines; it may be associated with the upregulation of genes related to T-cell activation in response to *Salmonella* challenge (van Hemert et al., 2000) or that conventional broilers have significantly higher concentrations of IgA and IgG on day 21 (Snyder et al., 2022).

The results from this experiment provide insight regarding the role of broiler genetic selection on the microbiome and how it may impact enteric colonization resistance. A breed effect on the ileal and cecal microbiome occurred between slow-growing and conventional broilers and the effect was dependent on the age and specific intestinal location, as a greater difference between the breeds was observed in the ileum in younger broilers, and in the cecum in older broilers. *Salmonella* Typhimurium challenge caused a shift in the microbial communities, and differences in bacterial relative abundances were observed in certain ages and intestinal regions. Some potentially beneficial microbes were depleted in broilers challenged with *Salmonella* Typhimurium. An interaction between broiler genetic line and *Salmonella* Typhimurium challenge was found and showed the microbiomes of the two different breeds were each negatively affected by *Salmonella* challenge, but at different ages. Results from the present study demonstrate the dynamic nature of the broiler microbiome and how pathogen exposure can result in temporary (or inconsistent across all ages) and localized changes to the intestinal microbiota.

## Data Availability

The datasets presented in this study can be found in online repositories. The names of the repository/repositories and accession number(s) can be found below: https://www.ncbi.nlm.nih.gov/, Bioproject PRJNA803251 and Biosamples SAMN25640069-SAMN25640548.
