# Supplementary material for: Ileal and cecal microbiota response to Salmonella Typhimurium challenge in conventional and slow-growing broilers
Source: Front Physiol. 2022 Oct 4;13:971255. doi: 10.3389/fphys.2022.971255 (PMC9577007; doi:10.3389/fphys.2022.971255)
Supplement: Supplementary file 1 [file Image1.pdf]

SUPPLEMENTARY INFORMATION

Ileal and cecal microbiota of conventional and slow-growing broilers and their response to *Salmonella* Typhimurium challenge

Tessa R. Sheets[1], Carmen L. Wickware[1], Ashlyn M. Snyder[2], Shawna L. Weimer[2,3], Timothy A. Johnson[1*]

[1]Department of Animal Sciences, Purdue University, West Lafayette, Indiana 47907-2050 USA
[2]Department of Animal and Avian Sciences, University of Maryland, College Park, MD 20742
[3]Current address: Department of Poultry Science, University of Arkansas, Fayetteville, AR

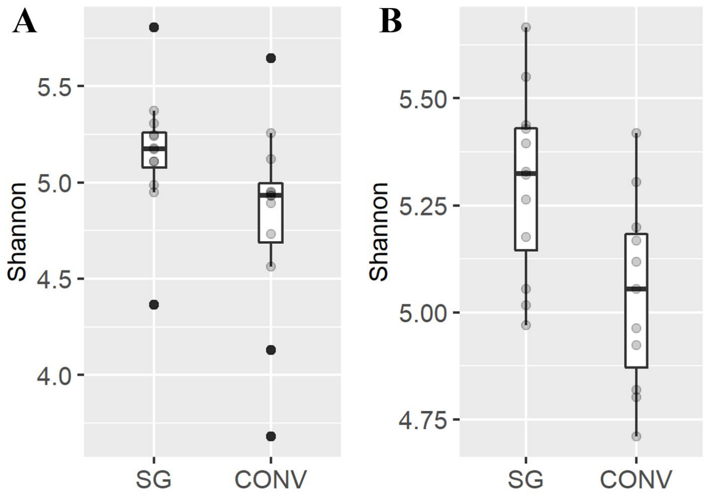

Figure S1: The effect of genetic line on the cecal Shannon diversity of 17 (**A**) and 21-day-old broilers (**B**). At both time points, slow-growing (SG) broilers were found to have significantly greater diversity compared to conventional (CONV) broilers.

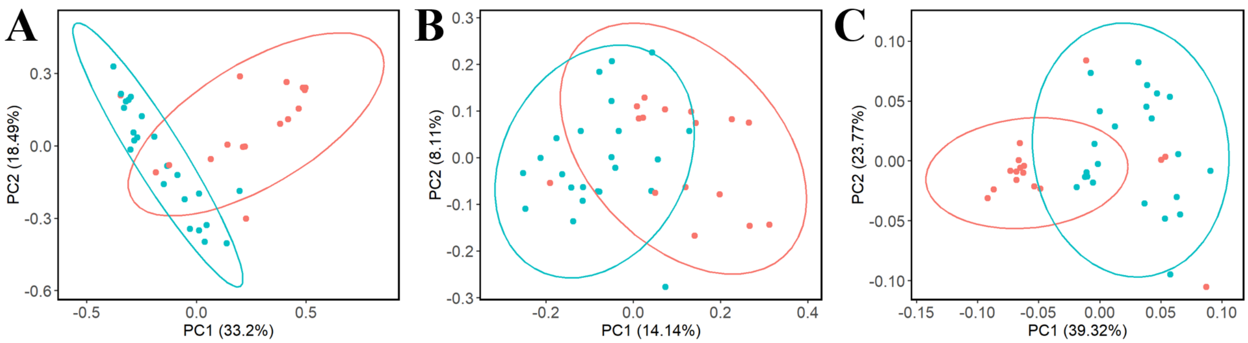

Figure S2: The effect of genetic line on beta diversity measures in the ileum of 7-day-old broilers. Significant dissimilarity was seen in Bray-Curtis (**A**), unweighted Unifrac (**B**), and weighted Unifrac (**C**). Red represents slow-growing (SG) broilers while blue represents conventional (CONV) broilers.

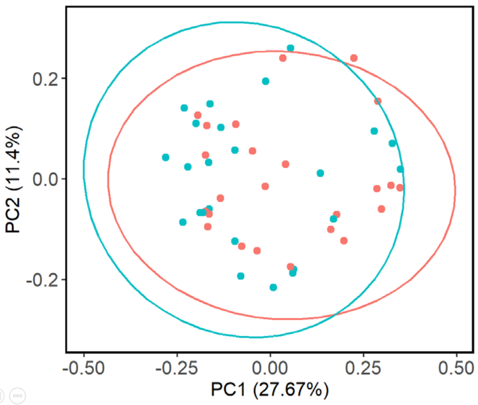

Figure S3: Significant dissimilarity was seen only in the Bray-Curtis measurement when evaluating the effect of genetic line on beta diversity measures in the cecum of 7-day-old broilers. Red represents slow-growing (SG) broilers while blue represents conventional (CONV) broilers.

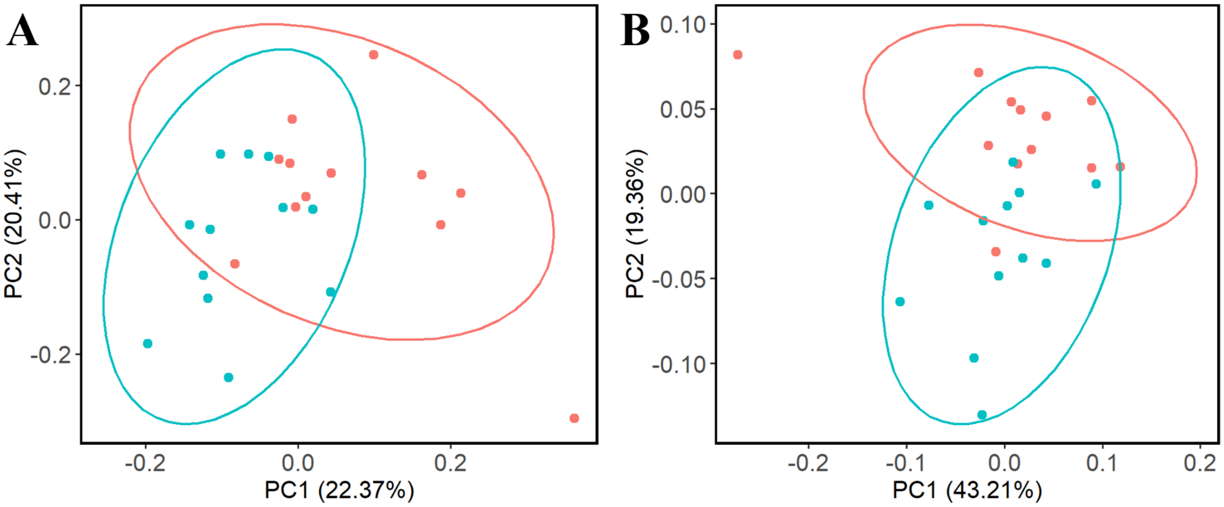

Figure S4: The effect of genetic line on beta diversity measures in the cecum of 13-day-old broilers. Significant dissimilarity was seen in Bray-Curtis (A) and weighted Unifrac (B). These were the only measures that were found to be significant on day 13. Red represents slow-growing (SG) broilers while blue represents conventional (CONV) broilers.

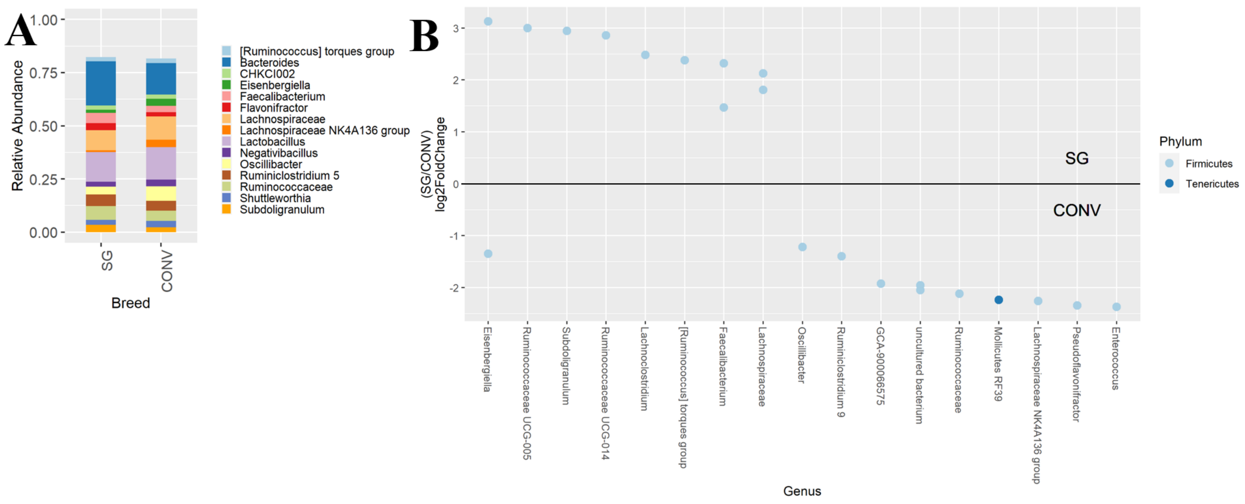

Figure S5: Relative abundance of genera in the cecum on day 13 that are present in greater than 2% of total taxa found within conventional (CONV) and slow-growing (SG) broilers (**A**). Differentially abundant amplicon sequence variants (ASVs) between broiler genetic lines in the cecum on day 13 (**B**). Significantly different ($p < 0.05$) ASVs are presented and organized by abundance within each group. ASVs enriched in SG broilers are indicated with a log 2-fold change > 0 while ASVs enriched in CONV broilers are indicated with a log 2-fold change of < 0. *Lachnospiraceae* NK4A136 and *Oscillibacter*, both short chain fatty acid producers, were significantly enriched in CONV broilers along with *Mollicutes* RF39 which has been found to be involved with energy harvesting and *Ruminiclostridium* 9 that may contribute to carbohydrate metabolism.

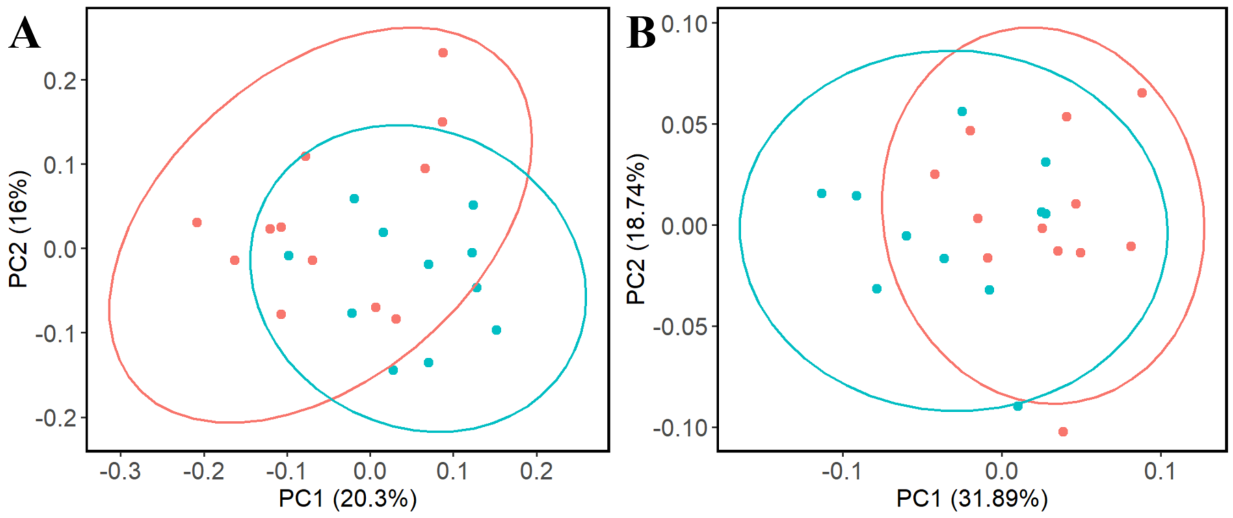

Figure S6: The effect of genetic line on beta diversity measures in the cecum of 21-day-old broilers. Significant dissimilarity was seen in Bray-Curtis (**A**) and weighted Unifrac (**B**). Red represents slow-growing (SG) broilers while blue represents conventional (CONV) broilers.

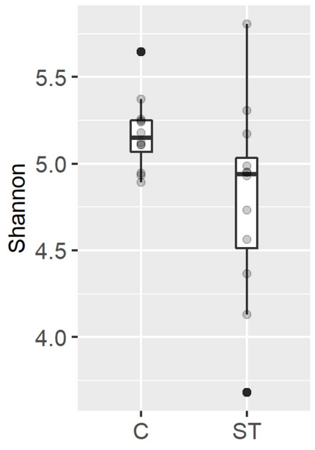

Figure S7: The effect of *Salmonella* challenge on the cecal Shannon diversity of 17-day-old broilers. Broilers challenged with *Salmonella* (ST) had significantly lower diversity compared to non-challenged (C) broilers.

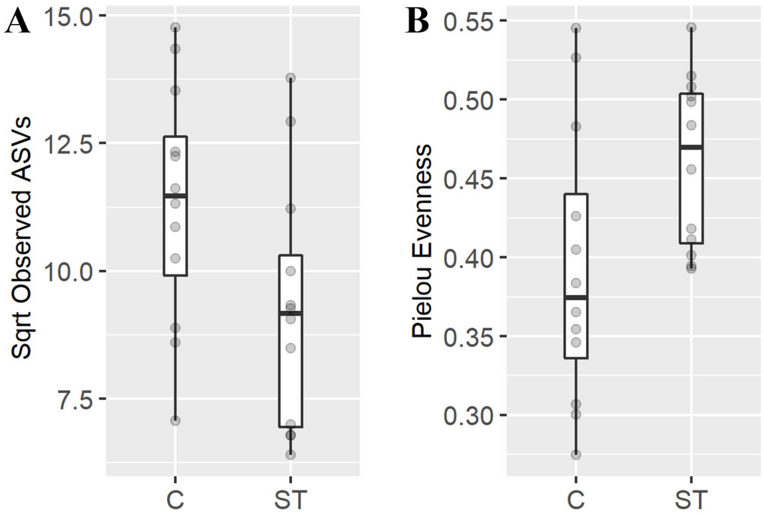

Figure S8: The effect of *Salmonella* challenge on the ileum of 21 (**A**) and 24-day-old broilers (**B**). Broilers challenged with *Salmonella* (ST) had significantly lower richness on day 21 but significantly higher evenness on day 24 compared to non-challenged (C) broilers when evaluating the ileum.

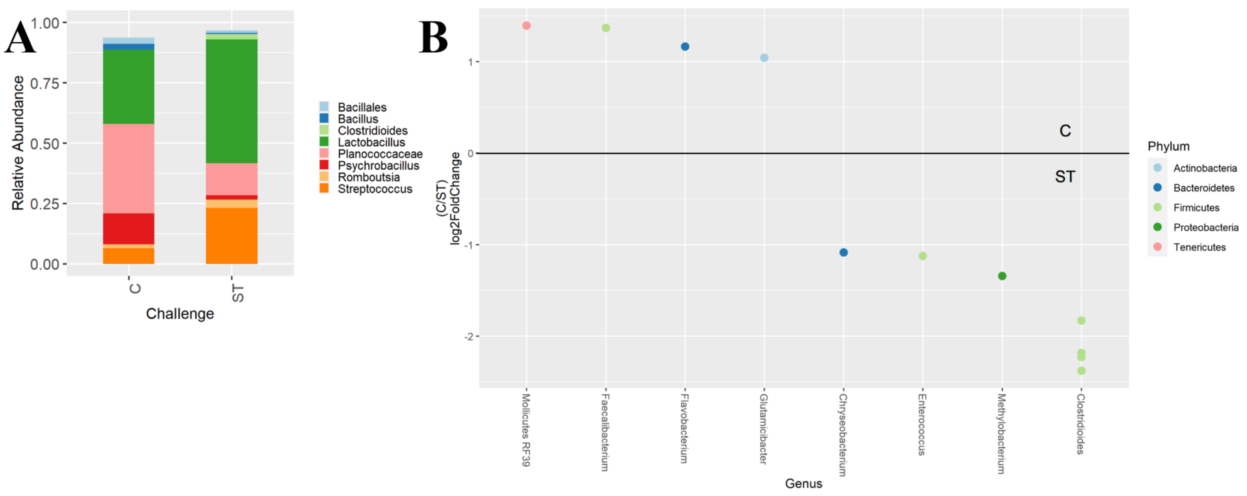

Figure S9: Relative abundance of genera in the ileum on day 24 that were present in greater than 2% of total taxa found within control (C) and *Salmonella* Typhimurium challenged (ST) broilers (**A**). Differentially abundant amplicon sequence variants (ASVs) between challenge groups in the ileum on day 24 (**B**). Significantly different ($p < 0.05$) ASVs are presented and organized by abundance within each group. ASVs enriched in non-challenged broilers are indicated with a log 2-fold change > 0 while ASVs enriched in challenged broilers are indicated with a log 2-fold change of < 0. Four ASVs from the *Clostridioides* genus were found to be significantly enriched (each approximately 4-fold) in the ileum of broilers 24 days of age that were challenged with *Salmonella*.

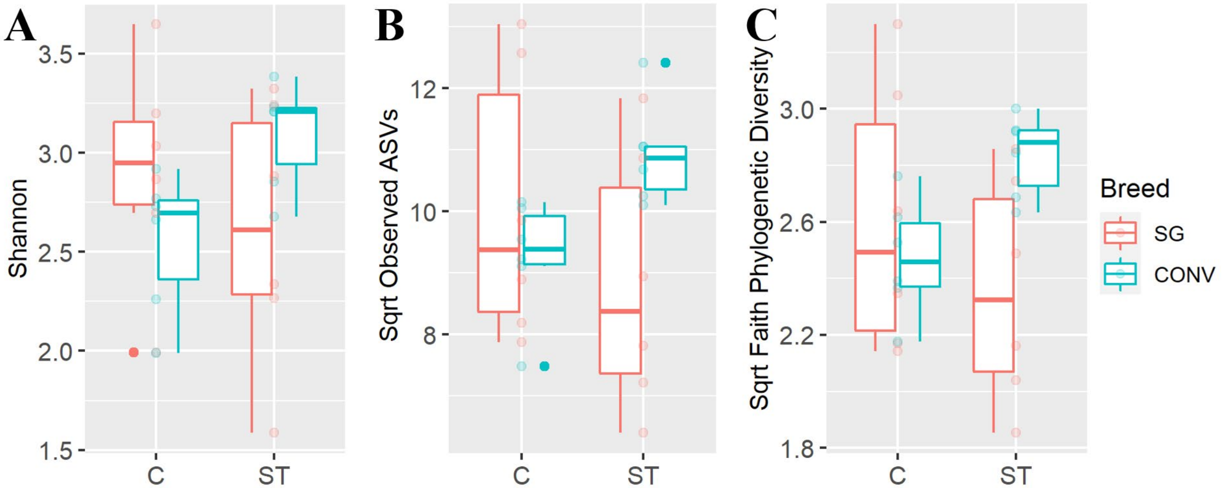

Figure S10: The interaction between of genetic line and *Salmonella* challenge on the ileal Shannon diversity (**A**), observed ASV richness (**B**), and Faith phylogenetic diversity (**C**) for 17-day-old broilers. No significant pairwise comparisons were found but challenged (ST) conventional (CONV) broilers consistently had higher alpha diversity measures compared to challenged (ST) slow-growing (SG) broilers.

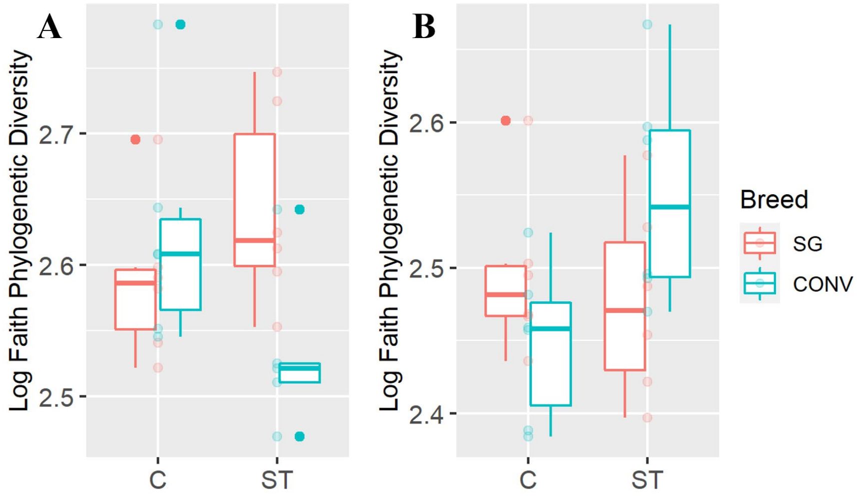

Figure S11: The interaction between of genetic line and *Salmonella* challenge on the cecal Faith phylogenetic diversity for broilers 21 (**A**) and 24 days of age (**B**). A significant pairwise comparison was not found in neither of the two datasets. Control (C) and *Salmonella* Typhimurium challenged (ST) broilers and conventional (CONV) and slow-growing (SG) broilers.

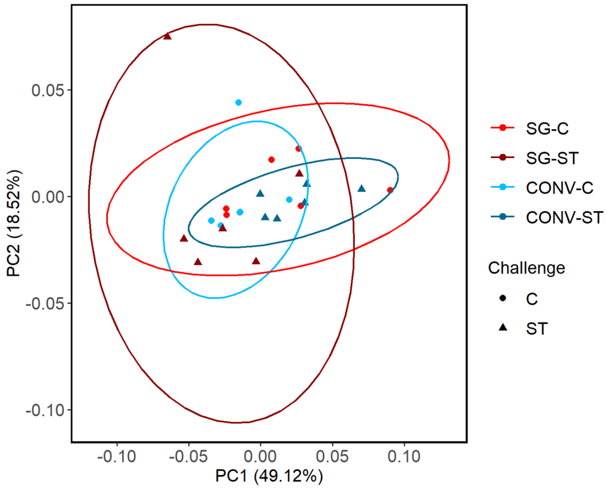

Figure S12: The interaction between genetic line and *Salmonella* challenge on the weighted Unifrac measure in the ileum of 17-day-old broilers. There was significant community dissimilarity between challenged (ST) and non-challenged (C) conventional (CONV) broilers which can be visualized by the dispersion between the two CONV (blue) ellipses. There is also a significant difference in treatment group clustering when evaluating weighted Unifrac between challenged slow-growing (SG) broilers and challenged CONV broilers, as indicated by the two darker ellipses.
